# Genome Sequence of Staphylococcus aureus Ex1, Isolated from a Patient with Spinal Osteomyelitis

**DOI:** 10.1128/genomeA.00623-18

**Published:** 2018-06-28

**Authors:** Helen Morcrette, Marina S. Morgan, Audrey Farbos, Paul O’Neill, Karen Moore, Richard W. Titball, David J. Studholme

**Affiliations:** a Biosciences, University of Exeter, Exeter, Devon, United Kingdom; b Department of Microbiology, Royal Devon and Exeter NHS Foundation Trust, Exeter, Devon, United Kingdom

## Abstract

Here, we present the genome sequence of Staphylococcus aureus Ex1, isolated in 2015 from a patient with spinal osteomyelitis at the Royal Devon and Exeter Hospital in the United Kingdom. The availability of the Ex1 genome sequence provides a resource for studying the basis for spinal infection and horizontal gene transfer in S. aureus.

## GENOME ANNOUNCEMENT

We have sequenced the genome of Staphylococcus aureus Ex1, isolated in April 2015 from a patient with spinal osteomyelitis in the Royal Devon and Exeter Hospital in the United Kingdom. This isolate’s *spa* type (1) is T3277 with the repeat succession 07-17-21-34-34-22-34.

We used an Illumina MiSeq instrument to generate 3,820,104 pairs of 300-bp reads corresponding to 779× average coverage, judged by aligning reads with the Burrows-Wheeler Aligner (BWA) software package (2) and measuring depth with Qualimap (3, 4). The reads were assembled into a 2,746,235-bp draft genome sequence consisting of 18 contigs, with an *N*_50_ length of 760,849 bp, using SPAdes version 3.11.1 (5). This assembled genome sequence was then annotated using the NCBI Prokaryotic Genome Annotation Pipeline (6).

VirulenceFinder (7) identified the following virulence factors encoded in this genome: aureolysin, serine protease SplA, serine protease SplE, staphylococcal complement inhibitor, staphylokinase, beta-hemolysin, gamma-hemolysin component B precursor, enterotoxin O, gamma-hemolysin component C, enterotoxin M, gamma-hemolysin chain II precursor, enterotoxin I, enterotoxin U, enterotoxin N, enterotoxin G, leukocidin E component, and leukocidin D component. Mykrobe Predictor (8) identified a *blaZ* gene, predicted to encode penicillin resistance. Also present are *tcaA*, *tcaB*, and *tcaR*, encoding teicoplanin resistance (9), and *fosA*, *fosB*, and *fosX*, predicted to encode fosfomycin resistance (10), as well as several putative efflux pumps whose substrates are not known.

Whole-genome sequence alignment with MUMmer4 (11) revealed very high similarity with S. aureus strain 10110051-4 (GenBank accession no. NGNL00000000), isolated from a surface swab from the International Space Station. These two genome sequences share 99.95% average nucleotide identity over at least 99.42% of their lengths. Alignment with Harvest (12) revealed that the genome of Ex1 contains a 300-kbp region that is nearly identical to those of the more distantly related genomes of S. aureus strains 21311 (GenBank accession no. JHPX00000000), 08-02119 (CP015645), and 510_SAUR (JVEG00000000) (13), suggesting a recombination event between the recent ancestors of these strains and those of Ex1, resulting in horizontal transfer of more than 10% of the genome. The availability of the Ex1 genome sequence provides a resource for studying the basis for spinal osteomyelitis infection and recombination in S. aureus.

### Accession number(s).

This whole-genome shotgun project has been deposited in DDBJ/ENA/GenBank under the accession no. QFZP00000000. The version described in this paper is the first version, QFZP01000000.
